# Impact of COVID-19 Pandemic on Pediatrics and Pediatric Transplantation Programs

**DOI:** 10.3389/fped.2020.612627

**Published:** 2020-12-10

**Authors:** Steven Lobritto, Lara Danziger-Isakov, Marian G. Michaels, George V. Mazariegos

**Affiliations:** ^1^Department of Pediatrics, Columbia University College of Physicians and Surgeons, New York, NY, United States; ^2^Division of Infectious Diseases, Cincinnati Children's Hospital Medical Center and University of Cincinnati, Cincinnati, OH, United States; ^3^Division of Infectious Diseases, UPMC Children's Hospital of Pittsburgh, Pittsburgh, PA, United States; ^4^Hillman Center for Pediatric Transplantation, UPMC Children's Hospital of Pittsburgh, Pittsburgh, PA, United States

**Keywords:** transplant volumes, treatment, telemedicine, comorbidities, return to school, resource utilization

## Abstract

COVID-19 has dramatically altered the health care landscape and disrupted global health and world economics in ways that are still being measured. Its impact on children with chronic conditions or those undergoing transplantation is evolving. The organ specific manifestations in children will be reviewed and treatment strategies outlined. The impact on pediatric transplantation in the United States over the initial 6 months of the pandemic has shown significant regional variation and lags persist in resumption of normal transplant activity, particularly for living related transplantation. Finally, guidelines regarding return to school will be discussed.

## Introduction

The novel coronavirus disease (COVID-19) pandemic, caused by severe acute respiratory syndrome coronavirus 2 (SARS-CoV-2), continues to effect humans worldwide. Whether directly infected or indirectly impacted by the economic implications, the psychosocial ramifications, the disruption of normal everyday interactions, the way we educate our children or the trust we have in our governmental representatives, our lives have all been impacted in some way. With over 33 million infected worldwide and over 1 million reported deaths the healthcare system has taken on the burden of reacting to this global health care crisis (https://www.worldometers.info/coronavirus/). Although new cases continue to mount daily, children account for an estimated 2–7.6% of reported SARS-CoV-2 cases, with death being uncommon(<0.1%) (1, 2).

SARS-CoV-2 is a single, positive-stranded RNA virus that replicates using a virally encoded RNA-dependent RNA polymerase. SARS-CoV-2 binds to, and is internalized into, target cells through angiotensin-converting enzyme (ACE) 2, which acts as a functional receptor (3, 4). The virus is transmitted on respiratory droplets via the mucosal surfaces of the eyes, nose or mouth and activates antiviral immune responses that can lead to uncontrolled inflammatory reactions characterized by marked pro-inflammatory cytokine release in patients severely affected leading to lymphopenia, lymphocyte dysfunction, and granulocyte and monocyte abnormalities (5). This in turn may lead to infections by microorganisms, septic shock, and severe multiple organ dysfunction (6). Given the importance of the immune response to infection we will discuss the significant impact on the transplant community.

## Disease Manifestation in Children and Organ Specific Impact With SARS-CoV-2/COVID-19

While covered in more detail in other chapters, it is important to transplantation to recognize that SARS-CoV-2 can impact multiple organs. In general, pediatric patients have less symptomatic disease compared to adults, but this is not always the case. When symptomatic, COVID-19 primarily manifests with respiratory symptoms and involvement of the pulmonary tree. Early epidemiology of SARS-CoV-2 infection in children in China showed that the vast majority were asymptomatic or had only mild to moderate symptoms. When symptomatic, COVID-19 was often indistinguishable from other acute respiratory viruses with fever, fatigue, myalgia, pharyngitis with or without fever (7, 8). Moderate symptoms included cough with or without wheezing and evidence of pneumonia with less frequent episodes of hypoxemia compared to adults. Severe disease occurred in <10% of cases but when present, the dyspnea and hypoxemia would generally develop around a week into the illness and deterioration could follow quickly. Children with more severe manifestation also were more likely to have gastrointestinal involvement. Aside from the gastrointestinal manifestations, extrapulmonary manifestations of COVID-19 have been noted broadly including acute kidney injury, hepatitis, neurologic manifestation and hematologic abnormalities.

Unique to children and young adults, a delayed illness with inflammation involving multiple systems of the body is recognized to occur and is called Multisystem Inflammatory Syndrome in Children (MIS-C). It manifests with fever and severe illness affecting at least two or more organ systems. including cardiac, renal, respiratory, gastrointestinal, neurological, dermatologic and/or hematologic (9–11). It was noted initially to have some overlapping features of Kawasaki Disease particularly including high fevers, rash, conjunctival involvement and myocardial involvement including coronary aneurysm. Ongoing research will help to clarify the immunologic basis and treatment for this disorder but in severe cases it is possible that some children may require transplantation if organ dysfunction fails to recover.

## The Impact of Comorbid Disease on SARS-CoV-2

Metanalyses of the published literature in adults and pediatric patients infected with SARS-CoV-2 have identified a number of medical conditions correlating with disease severity and being admitted to the intensive care setting (12–14). Some of the factors identified include diabetes, hypertension, coronary artery disease/cardiovascular disease, chronic pulmonary disease, malignancy, chronic kidney disease, older age and male gender although the heterogeneity between studies varied substantially. The comorbidities leading to more severe disease in children are less understood with obesity being reported most commonly (15–17). In general, pediatric patients with kidney failure (chronic kidney disease, stage 5) supported by hemodialysis or home peritoneal dialysis are at significant risk for experiencing infectious diseases such as COVID-19 because of their compromised immune system and their frequent exposure to the hospital setting. The factors potentially contributing to the risk of SARS-CoV-2 infection in the pediatric chronic kidney disease population include: (a) compromised immune system (the result of long-term malnutrition, uremia, and/or immunosuppressants); (b) close proximity to other patients during treatment in a confined HD unit; (c) frequent contact with healthcare workers, who may be asymptomatic but infected while caring for a variety of other patients; (d) a need for the presence of parents or other relatives during the treatment, which increases the risk of cluster infection; and (e) non-adherence to, or a break in implementation of recommended infection prevention practices (18). Interestingly, early case reports of SARS-CoV-2 infection outcomes in children with chronic kidney disease conclude that affected patients have a similar clinical outcome as in healthy children of the same age even if immunosuppressed; however, special attention must be paid to fluid management and drug dose adjustment (19).

Patients with advanced liver disease and those after liver transplantation represent vulnerable populations with an increased risk of infection and/or a potentially severe course of COVID-19 (20). Given that the ACE2 receptor is present in biliary and liver epithelial cells, the liver is a potential target for SARS-CoV-2 infection. Fortunately, as noted above, severe COVID-19 is uncommon in children (21, 22). Most children have mild symptoms, though hepatic manifestations can be significant (2, 23–27). Certainly, some children require hospitalization for severe illness with over half having an underlying medical condition, most commonly obesity (1, 15, 28–30). Likewise, MIS-C can include significant hepatic injury and even acute liver failure (9, 31–33). In these cases, liver disease may be secondary to drug-induced liver injury, cytokine storm and/or pneumonia-associated hypoxia.

One additional population at risk includes patients with inborn metabolic diseases such as defects of amino acid, urea cycle, organic acid, carbohydrate and energy metabolism, and organelle disorders (lysosomal storage diseases and peroxisomal diseases). Most of these patients require meticulous dietary and therapy interventions as well as close monitoring of their clinical status. Indeed, some patients are likely to be particularly fragile and at risk of life-threatening acute metabolic decompensation in case of SARS-CoV-2 infection. The conditions at greatest risk include patients with defects of amino acid and organic acid metabolism, urea cycle defects, and disorders of carbohydrate and energy metabolism. Other patients, such as those with substantial neuromuscular involvement, such as Pompe disease, are likely susceptible to greater risks in case of respiratory disease and need for ventilatory assistance. Fortunately, to date single center reports show that through preventive measures and adequate resource allocation major morbidity and mortality can be avoided in this at-risk population (34). The common challenge in the management of pediatric patients with chronic underlying conditions is the shift in healthcare resource allocation necessary to deal with the pandemic that may negatively impact the care of patients that continue to require intensive medical attention.

## SARS-CoV-2 Infection in Children Receiving Immunosuppressive Treatment

As the pandemic continues the number of transplanted patients, both children and adults and the number of patients immunosuppressed for other autoimmune conditions or cancers is steadily rising. Initial reports of outcomes in solid organ transplant recipients suggested a more severe course, albeit with limitations on testing of less severe cases (35, 36). Immunosuppressive medications predispose to certain viral pathogens including herpesviruses (CMV, EBV, HSV, and VZV) BK virus, adenovirus, norovirus, influenza, and respiratory syncytial virus (37, 38). This does not seem to be the case for infections caused by the coronavirus family to date (22). In fact, morbidity seems more related to exaggerated host immune responses than to direct viral cytopathic injury (5). Children seem to be less at risk of serious consequence than adults but still pose an infectious risk to others. Although there are no well-controlled randomized studies to support general recommendations, experiences with other viral infections (hepatitis C, cytomegalovirus) suggest that minimizing or eliminating drugs that are associated with leukopenia may be prudent (39). An analysis of various databases containing immunosuppressed transplant recipients suggest that chronic immunosuppression could exert a protective effect against the most severe forms of COVID-19 and complete withdrawal of immunosuppression may not be useful (40). A final point regarding immunosuppression is the importance of recognizing possible drug interactions, especially in the case of tacrolimus, with some of the treatments with antiviral effect given in the context of COVID-19 (lopinavir/ritonavir, azithromycin) (41).

## Treatment of Covid in Children

Limited data exist regarding effective treatment strategies for SARS-CoV-2 in pediatric patients. Collaboratives of pediatric providers, the National Institutes of Health (NIH) and the Infectious Diseases Society of America (ISDA) have issued treatment recommendations predominantly extrapolated from adult studies (42–44). There are currently no FDA approved therapeutic agents for the treatment of COVID-19 in pediatric patients, although both remdesivir and SARS-CoV-2 rich convalescent plasma are available through an emergency use authorization. A recent report from a collaborative of pediatric providers suggests administration of 5 days of remdesivir, preferably as part of enrollment in a prospective clinical trial, for pediatric patients requiring supplemental oxygen (43). The NIH suggests consideration of remdesivir for pediatric patients with severe disease and both the IDSA and NIH endorse dexamethasone and remdesivir in adult patients with hypoxemia (44, 45). Neither the NIH nor the IDSA suggest use of convalescent plasma outside of the context of a clinical trial (44, 45). Several additional therapies are under investigation including antivirals (favipiravir) and immunomodulatory agents (tocilizumab, IL-1 inhibitors, Janus kinase inhibitors and complement inhibitors) but the majority of studies focus on adult patients and do not include pediatric solid organ transplant (SOT) recipients.

## Impact on Transplant Activity

The global pandemic has wide-ranging implications to transplantation programs beginning with the impact on donor and recipients, health resource utilization and strategies to mitigate risk during the pandemic. There is a ripple effect on post-transplant life including return to school, routine post-transplant follow-up, and vaccine development and its implications.

Global recommendations continue to evolve but early on demonstrated a staged approach to transplant activity based on recipient illness and urgency as well as system resource utilization requirements (46). Guidelines emerged quickly regarding optimal donor screening based on nasopharyngeal specimen NAT testing and were utilized in the United States (U.S.) sporadically initially but almost universally by late March 2020.

Initial impact on transplantation center practices and policies have for the most part been focused on adult transplantation. Boyarsky and colleagues surveyed U.S. centers between March 24- March 31, 2020 and demonstrated early impact of complete suspension of living donor kidney transplantation in 71.8% of centers and live donor liver transplantation in 67.7% of respondents with restrictions being linked to regional incidences of COVID-19 (47). Experience with resumption of activity have been documented in several series including United Kingdom (UK), U.S. and other centers (48). Recently, the US experience with liver transplantation compared waitlist outcomes and transplant activity between 3/15/2020 and 8/31/2020 to historical trends. While new listings during the initial 6 weeks of the pandemic were decreased by 11%, by August, waitlist outcomes were occurring at expected rates and deceased donor transplants were 13% more across all incidences (49).

**Figure 1:**
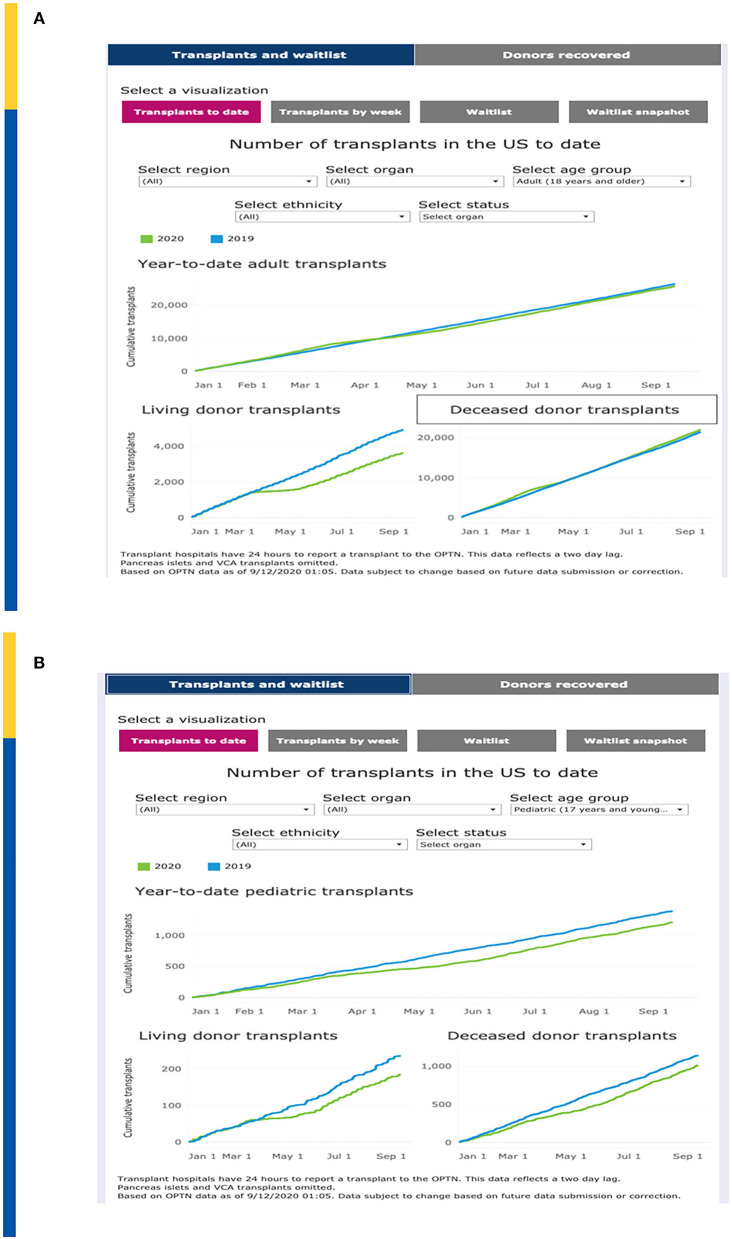
**(A)** Impact on Total Transplant Activity in U.S. To Date. **(B)** Impact on Pediatric Transplant Activity in U.S. To Date. Adapted from: https://unos.org/covid.

United Network for Organ Sharing (UNOS) has continuously updated transplant and waitlist data during the COVID-19 pandemic. Year-to-date transplants as of 9/10/2020 demonstrate a total organ transplant count across all ages of 26,724 as compared to 27,592 over the same time period in 2019, a decrease of 3.4% (Figure 1A). This relatively small decrease demonstrates a significant resiliency of the transplant enterprise in the U.S. but also significant variation by geography and by transplant type. For example, transplant activity in the Northeast and Mid-Atlantic regions has not caught up to 2019 levels whereas as of the writing of this manuscript, transplant activity in the southeastern U.S. is unchanged between 2019 and 2020.

Of concern, the impact on pediatric transplantation activity has demonstrated some significant differences that demonstrate both opportunities as well as challenges. Overall, pediatric transplantation is down approximately 10% in 2020. Compared to 1,375 pediatric transplants performed by early September 2019, as of 9/10/2020 there were only 1,202 pediatric transplants (Figure 1B). The major decrease is seen with living donor transplantation compared to deceased donor transplantation which may reflect a step wise reduction in activity, beginning with “elective” living donor transplants during the early phases of the pandemic. Nonetheless, the curves demonstrated a persistent lag in catchup activity through September 2020. However, in some regions such as the mid-Atlantic, living donation has mitigated the decrease of available deceased donors in the same region. The regional differences are also impacted by the nearly simultaneous advent of acuity circle distribution in the US which was instituted on February 4, 2020.

**Figure 2:**
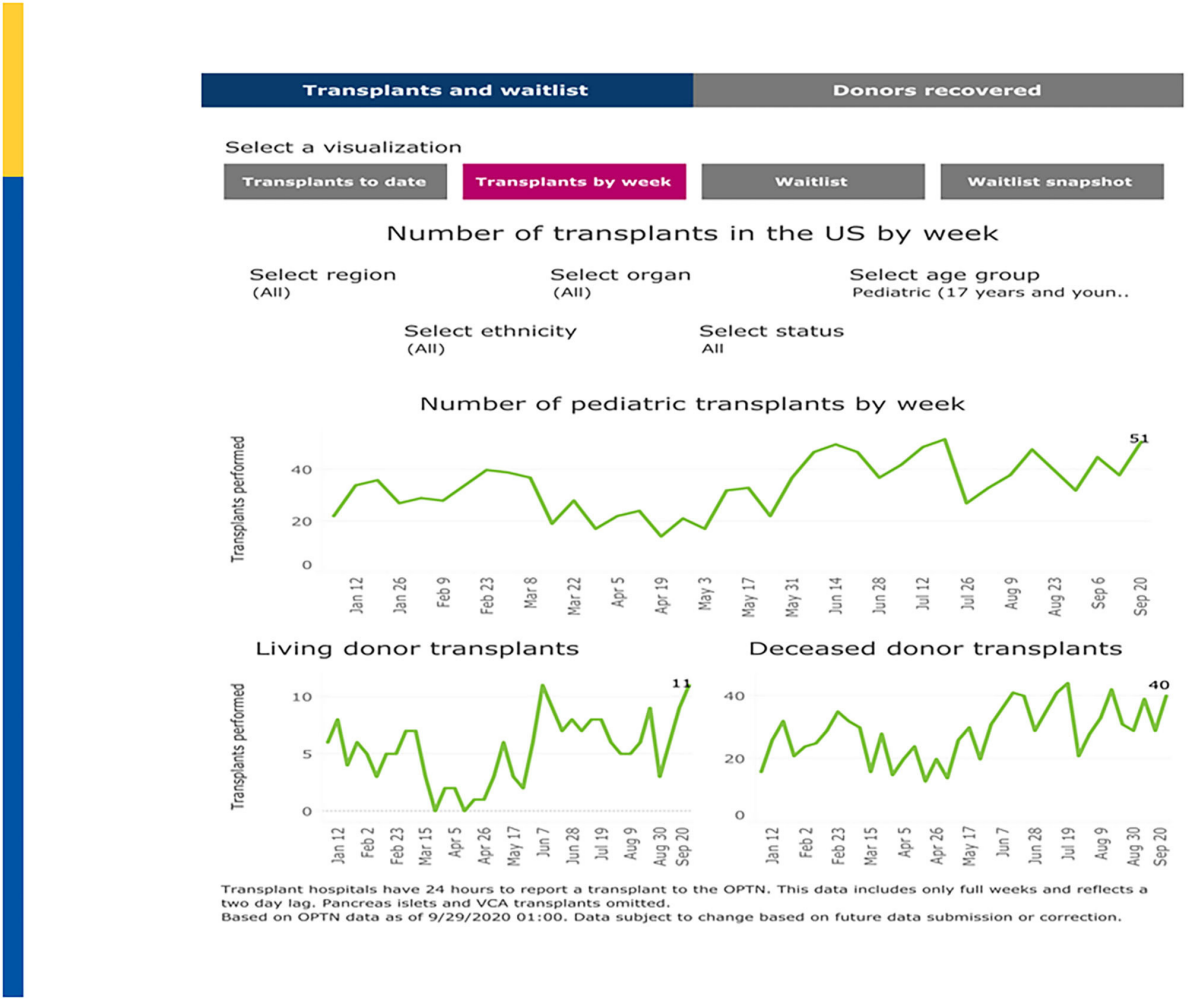
Pediatric Transplants by Week (January–September 2020). Adapted from: https://unos.org/covid.

The U.S. impact on transplantation in children began to noticeably diverge from 2019 levels in mid-March, 2020, corresponding with the March 11 declaration of the global COVID-19 pandemic by the World Health Organization (WHO) and the early regional outbreaks in the northeastern and northwestern U.S. For example, living donor transplants dramatically fell on the week of March 15 before beginning to rebound in late April. Deceased donor transplants in children fell earlier, on the week of March 8 and began to rebound earlier. As shown in Figure 2, pediatric transplant activity by week is now approaching pre-pandemic levels (50).

**Figure 3:**
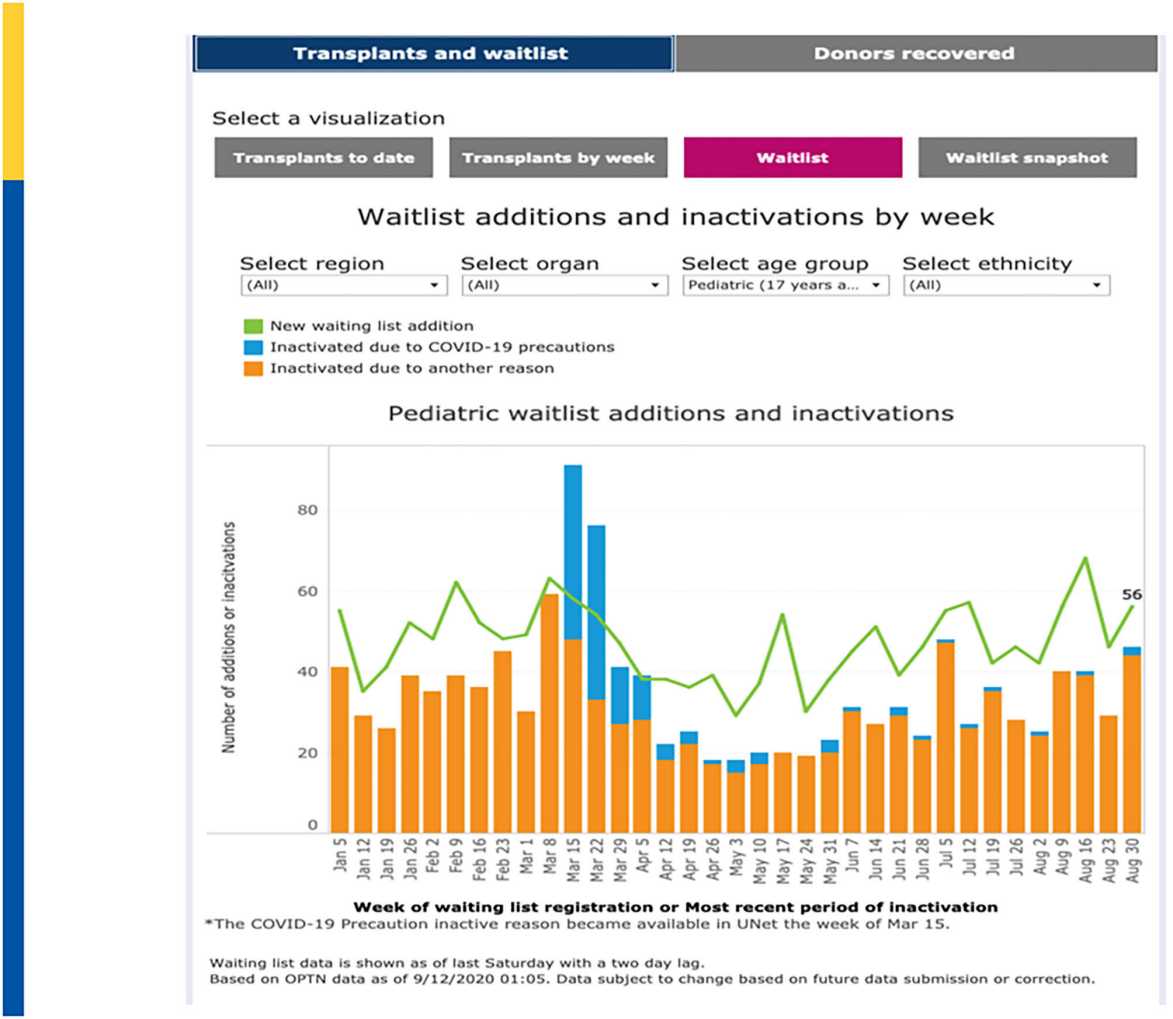
Waitlist Additions & Inactivations by Week. Adapted from: https://unos.org/covid.

New wait list additions fell from a high of 1,435 additions on the week of March 1–March 7, 2020 to a low in 2020 of only 789 additions the week of May 3–May 9, 2020 before rebounding back to 1,294 additions on the week of August 30–September 5, 2020 (Figure 3). March 22–March 28, 2020 showed the greatest number of “inactivations” due to COVID-19 “precautions” during which time 72% of inactivations where coded for this reason. Since May 24, 2020, inactivations due to COVID-19 precautions have averaged approximately 2–3% (50). A recent analysis in pediatric kidney transplantation similarly emphasized a significant decrease in DDKT and LDKT of 47% and 82% compared to expected events. However, by May 2020, the transplant activity was approaching prepandemic levels (51).

## Telemedicine-Now and Moving Forward

One of the potentially enduring changes that the COVID pandemic has actuated has been the institution of telehealth. The COVID−19 Telehealth program provided $200 million in funds as part of the Coronovirus Aid, Relief, and Economic Security (CARES) Act. The program provided immediate support to health care providers responding to the pandemic by providing fully funded telecommunication services, information dissemination and devices necessary to provide connected care services (52). Several of the awardees including UPMC Children's Hospital of Pittsburgh, Children's Hospital Colorado, Mt. Sinai Hospital NY and Cincinnati Children's Hospital could continue to provide transplant follow-up to significant pediatric populations.

**Figure 4:**
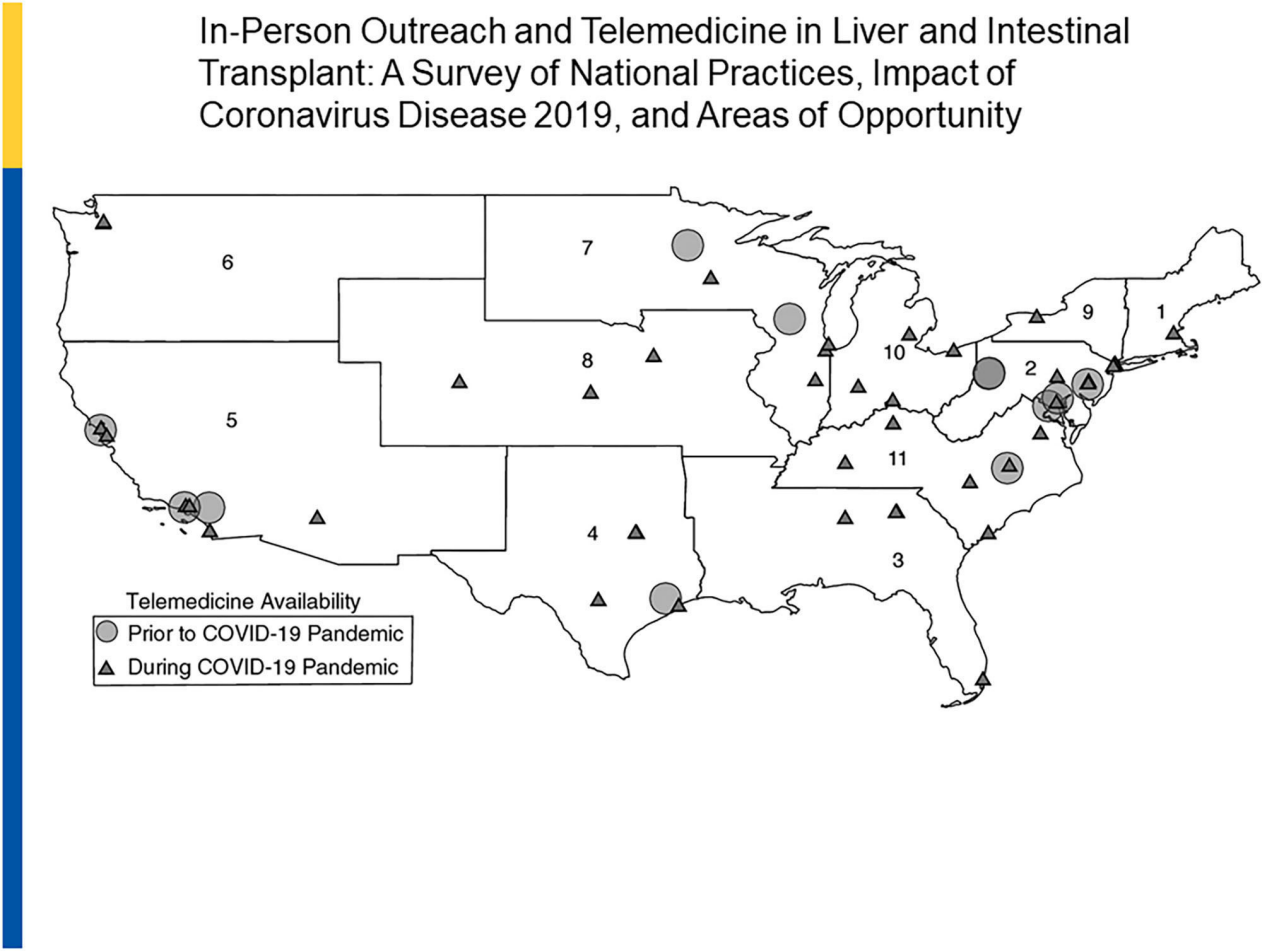
Telemedicine in Liver and Intestine Transplant: 2019 vs. 2020. Reprinted with permission: Liver Transplantation, First publisher: 09 August 2020, doi: 10.1002/lt.25868.

In one adult-based survey of U.S. based adult transplant programs by UNOS regions, a notable change between pre- and post -COVID telehealth utilization was documented. In 2019, of a total of 73 responding centers, 16% used telemedicine as compared to in-person visits in outreach clinics. In 2020, 54 of the 55 (98%) originally surveyed programs across all 11 UNOS regions now used telemedicine. Telemedicine is now being used by programs for transplant evaluations (65%), wait list management (58%), and post-transplant care (98%). In addition, 82% of centers used a combination of live video and phone strategies (53) (Figure 4).

## SARS-CoV-2 and School in Sot Recipients

Recommendations around in-person school attendance for SOT recipients in the era of SARS-CoV-2 remain challenging with little definitive data for guidance. Best practice development has focused on the epidemiology and impact of SARS-CoV-2 in SOT patients, community penetrance of COVID-19 disease and school-specific infection prevention intervention implementation in a consensus document recently published (54). Although pediatric SOT recipients are at increased risk for severe manifestations of common infections (55), case reports and case series of SARS-CoV-2 infection in pediatric SOT emerging thus far suggest pediatric SOT recipients are at similar risk and present with a similar spectrum of infection as other children (19, 56–59); however data are significantly limited and caution is still recommended. For example, an evaluation of the UK Transplant Registry linked with the National Health Service Digital Tracing Services identified only three cases of SARS-CoV-2 among 1,703 pediatric SOT recipients (0.5%) and no deaths occurred during a 4-month period during the height of the first wave of the pandemic in the UK (60). Delineation of risk for individual SOT recipients is proposed based on host factors such as time from transplant, maintenance immunosuppressive regimen, recent augmentation of immunosuppression and developmental capability of participating in infection prevention strategies to reduce risk, such as social distancing and mask wearing. Community prevalence is a non-specific marker of general risk that could be considered especially during periods of high transmission rates within a community. School-based infection prevention interventions support limiting transmission, evidenced by the impact of mask-wearing, physical distancing, hand hygiene, frequent disinfection and exclusion of ill students and staff as suggested by the Centers for Disease Control and Prevention and the American Academy of Pediatrics (61–63). Certainly the balance between risk for transmission, ability to limit exposure and the social and emotional development of pediatric SOT recipients should be weighed and decision-making will likely require flexibility and fluidity depending on the individual, community and school-based factors as the pandemic continues (54).

## SARS-CoV-2 and Safety Measures Necessary for Program/Clinic Reopening

In order to ensure patient and staff safety with the goal of achieving pre-COVID clinical and transplant activity, centers have adopted some general practices with the help of societal guidance (41). These practices include healthcare staff and patient training, body temperature monitoring, appropriate use/availability of personal protective equipment, universal masking and face shielding, frequent disinfection, spaced scheduling, hand hygiene, remote medical care, limiting accompanying persons, altering patient clinic flow through and pre-procedural SARS-CoV-2 nasal or nasopharyngeal PCR testing. Other prudent measures include conducting medical and surgical transplant rounds with the minimum number of personnel needed to provide care at a given time, rotating in-person office/clinical staff, limiting the number of team members who enter a patient's room for patient examinations, encounters and consultations and conducting virtual multidisciplinary rounds with dietary, pharmacy, social work, and care coordination staff. Waiting rooms for clinic visits, procedures, laboratory sampling and radiologic procedures have been eliminated or adapted to accommodate adequate patient spacing. Although initially suspending non-urgent procedures and in some cases living donor transplantations as above, centers should be able to offer full services with adequate safety measures in place. One of the greatest challenges lies with re-establishing patient and family confidence in the implementation and effectiveness of imposed safety measures. Nosocomial SARS-CoV-2 infection that was once common is quite rare and practically non-existent. Through the use of educational webinars, codified hospital clinical policies and visibly apparent safety measures, we can once again welcome patients back to the hospital when deemed essential.

## Data Availability Statement

The original contributions generated for this study are included in the article/supplementary material, further inquiries can be directed to the corresponding author/s.

## Author Contributions

All authors contributed equally to the content, design, and review of the manuscript.

## Conflict of Interest

The authors declare that the research was conducted in the absence of any commercial or financial relationships that could be construed as a potential conflict of interest.

## References

[1] Kim L Whitaker M O'Halloran A Kambhampati A Chai SJ Reingold A. Hospitalization rates and characteristics of children aged <18 years hospitalized with laboratory-confirmed COVID-19 - COVID-NET, 14 States, March 1-July 25, (2020). MMWR Morb Mortal Wkly Rep. (2020) 69:1081–8. 10.15585/mmwr.mm6932e3 32790664 PMC7440125

[2] Wu Z McGoogan JM. Characteristics of and important lessons from the coronavirus disease 2019 (COVID-19) outbreak in China: summary of a report of 72314 cases from the Chinese Center for disease control and prevention. JAMA. (2020) 323:1239–42. 10.1001/jama.2020.2648 32091533

[3] Lan J Ge J Yu J Shan S Zhou H Fan S. Structure of the SARS-CoV-2 spike receptor-binding domain bound to the ACE2 receptor. Nature. (2020) 581:215–20. 10.1038/s41586-020-2180-5 32225176

[4] Li W Moore MJ Vasilieva N Sui J Wong SK Berne MA. Angiotensin-converting enzyme 2 is a functional receptor for the SARS coronavirus. Nature. (2003) 426:450–4. 10.1038/nature02145 14647384 PMC7095016

[5] Ovsyannikova IG Haralambieva IH Crooke SN Poland GA Kennedy RB. The role of host genetics in the immune response to SARS-CoV-2 and COVID-19 susceptibility and severity. Immunol Rev. (2020) 296:205–19. 10.1111/imr.12897 32658335 PMC7404857

[6] Yang L Liu S Liu J Zhang Z Wan X Huang B. COVID-19: immunopathogenesis and Immunotherapeutics. Signal Transduct Target Ther. (2020) 5:128. 10.1038/s41392-020-00243-2 32712629 PMC7381863

[7] Dong Y Mo X Hu Y Qi X Jiang F Jiang Z. Epidemiology of COVID-19 among children in China. Pediatrics. (2020) 145:e20200702. 10.1542/peds.2020-0702 32179660

[8] Liguoro I Pilotto C Bonanni M Ferrari ME Pusiol A Nocerino A. SARS-COV-2 infection in children and newborns: a systematic review. Eur J Pediatr. (2020) 179:1029–46. 10.1007/s00431-020-03684-7 32424745 PMC7234446

[9] Verdoni L Mazza A Gervasoni A Martelli L Ruggeri M Ciuffreda M. An outbreak of severe Kawasaki-like disease at the Italian epicentre of the SARS-CoV-2 epidemic: an observational cohort study. Lancet. (2020) 395:1771–8. 10.1016/S0140-6736(20)31103-X 32410760 PMC7220177

[10] Riphagen S Gomez X Gonzalez-Martinez C Wilkinson N Theocharis P. Hyperinflammatory shock in children during COVID-19 pandemic. Lancet. (2020) 395:1607–8. 10.1016/S0140-6736(20)31094-1 32386565 PMC7204765

[11] Feldstein LR Rose EB Horwitz SM Collins JP Newhams MM Son MBF. Multisystem inflammatory syndrome in children US, and adolescents. N Engl J Med. (2020) 383:334–46. 10.1056/NEJMoa2021680 32598831 PMC7346765

[12] Jutzeler CR Bourguignon L Weis CV Tong B Wong C Rieck B. Comorbidities, clinical signs and symptoms, laboratory findings, imaging features, treatment strategies, and outcomes in adult and pediatric patients with COVID-19: a systematic review and meta-analysis. Travel Med Infect Dis. (2020) 37:101825. 10.1016/j.tmaid.2020.101825 32763496 PMC7402237

[13] Liu H Chen S Liu M Nie H Lu H. Comorbid chronic diseases are strongly correlated with disease severity among COVID-19 patients: a systematic review and meta-analysis. Aging Dis. (2020) 11:668–78. 10.14336/AD.2020.0502 32489711 PMC7220287

[14] Emami A Javanmardi F Pirbonyeh N Akbari A. Prevalence of underlying diseases in hospitalized patients with COVID-19: a systematic review and meta-analysis. Arch Acad Emerg Med. (2020) 8:e35. 10.1371/journal.pone.0241265 32232218 PMC7096724

[15] Zachariah P Johnson CL Halabi KC Ahn D Sen AI Fischer A. Epidemiology, clinical features, and disease severity in patients with coronavirus disease 2019 (COVID-19) in a children's hospital in New York City, New York. JAMA Pediatr. (2020) 174:e202430. 10.1001/jamapediatrics.2020.2430 32492092 PMC7270880

[16] Gao F Zheng KI Wang XB Sun QF Pan KH Wang TY. Obesity is a risk factor for greater COVID-19 severity. Diabetes Care. (2020) 43:e72–4. 10.2337/dc20-0682 32409499

[17] Korakas E Ikonomidis I Kousathana F Balampanis K Kountouri A Raptis A. Obesity and COVID-19: immune and metabolic derangement as a possible link to adverse clinical outcomes. Am J Physiol Endocrinol Metab. (2020) 319:E105–9. 10.1152/ajpendo.00198.2020 32459524 PMC7322508

[18] Shen Q Wang M Che R Li Q Zhou J Wang F. Consensus recommendations for the care of children receiving chronic dialysis in association with the COVID-19 epidemic. Pediatr Nephrol. (2020) 35:1351–7. 10.1007/s00467-020-04555-x 32333285 PMC7181108

[19] Melgosa M Madrid A Alvarez O Lumbreras J Nieto F Parada E. SARS-CoV-2 infection in Spanish children with chronic kidney pathologies. Pediatr Nephrol. (2020) 35:1521–4. 10.1007/s00467-020-04597-1 32435879 PMC7237873

[20] Boettler T Newsome PN Mondelli MU Maticic M Cordero E Cornberg M Care of patients with liver disease during the COVID-19 pandemic: EASL-ESCMID position paper. JHEP Rep. (2020) 2:100113 10.1016/j.jhepr.2020.100113 32289115 PMC7128473

[21] Lu X Zhang L Du H Zhang J Li YY Qu J SARS-CoV-2 infection in children. N Engl J Med. (2020) 382:1663–5. 10.1056/NEJMc2005073 32187458 PMC7121177

[22] D'Antiga L. Coronaviruses and immunosuppressed patients: the facts during the third epidemic. Liver Transpl. (2020) 26:832–4. 10.1002/lt.25756 32196933

[23] Patel KP Patel PA Vunnam RR Hewlett AT Jain R Jing R. Gastrointestinal, hepatobiliary, and pancreatic manifestations of COVID-19. J Clin Virol. (2020) 128:104386. 10.1016/j.jcv.2020.104386 32388469 PMC7189193

[24] Redd WD Zhou JC Hathorn KE McCarty TR Bazarbashi AN Thompson CC. Prevalence and characteristics of gastrointestinal symptoms in patients with severe acute respiratory syndrome Coronavirus 2 infection in the United States: a multicenter cohort study. Gastroenterology. (2020) 159:765–7. 10.1053/j.gastro.2020.04.045 32333911 PMC7195377

[25] Xu L Liu J Lu M Yang D Zheng X. Liver injury during highly pathogenic human coronavirus infections. Liver Int. (2020) 40:998–1004. 10.1111/liv.14435 32170806 PMC7228361

[26] Zhang C Shi L Wang FS. Liver injury in COVID-19: management and challenges. Lancet Gastroenterol Hepatol. (2020) 5:428–30. 10.1016/S2468-1253(20)30057-1 32145190 PMC7129165

[27] Henry BM Benoit SW de Oliveira MHS Hsieh WC Benoit J Ballout RA. Laboratory abnormalities in children with mild and severe coronavirus disease 2019 (COVID-19): a pooled analysis and review. Clin Biochem. (2020) 81:1–8. 10.1016/j.clinbiochem.2020.05.012 32473151 PMC7251358

[28] Centers For Disease Control and Prevention. Demographic Trends of COVID-19 cases and deaths in the US reported to CDC. (2020). Available onlne at: https://www.cdc.gov/covid-data-tracker/index.html#demographics (accessed September 29, 2020).

[29] Shekerdemian LS Mahmood NR Wolfe KK Riggs BJ Ross CE McKiernan CA. Characteristics and outcomes of children with coronavirus disease 2019 (COVID-19) infection admitted to US and Canadian pediatric intensive care units. JAMA Pediatr. (2020) 174:868–73. 10.1001/jamapediatrics.2020.1948 32392288 PMC7489842

[30] Gotzinger F Santiago-Garcia B Noguera-Julian A Lanaspa M Lancella L Calo Carducci FI. COVID-19 in children and adolescents in Europe: a multinational, multicentre cohort study. Lancet Child Adolesc Health. (2020) 4:653–61. 10.1016/S2352-4642(20)30177-2 32593339 PMC7316447

[31] Godfred-Cato S Bryant B Leung J Oster ME Conklin L Abrams J. COVID-19-associated multisystem inflammatory syndrome in children - United States, March-July 2020. MMWR Morb Mortal Wkly Rep. (2020) 69:1074–80. 10.15585/mmwr.mm6932e2 32790663 PMC7440126

[32] Cantor A Miller J Zachariah P DaSilva B Margolis K Martinez M. Acute hepatitis is a prominent presentation of the multisystem inflammatory syndrome in children: a single-center report. Hepatology. (2020) 72:1522–27. 10.1002/hep.31526 32810894 PMC7655704

[33] Centers For Disease Control and Prevention Information for Healthcare Providers about Multisystem Inflammatory Syndrome in Children (IMIS-C). (2020). Available online at: https://www.cdc.gov/mis-c/hcp/ (accessed September 29, 2020).

[34] Brunetti-Pierri N Fecarotta S Staiano A Strisciuglio P Parenti G. Ensuring continuity of care for children with inherited metabolic diseases at the time of COVID-19: the experience of a metabolic unit in Italy. Genet Med. (2020) 22:1178–80. 10.1038/s41436-020-0831-4 32409735 PMC8629446

[35] Pereira MR Mohan S Cohen DJ Husain SA Dube GK Ratner LE. COVID-19 in solid organ transplant recipients: initial report from the US epicenter. Am J Transplant. (2020) 20:1800–8. 10.1111/ajt.15941 32330343 PMC7264777

[36] Lee BT Perumalswami PV Im GY Florman S Schiano TD Group CS. COVID-19 in liver transplant recipients: an initial experience from the US epicenter. Gastroenterology. (2020) 159:1176–8 e2. 10.1053/j.gastro.2020.05.050 32442561 PMC7237372

[37] Kaltsas A Sepkowitz K. Community acquired respiratory and gastrointestinal viral infections: challenges in the immunocompromised host. Curr Opin Infect Dis. (2012) 25:423–30. 10.1097/QCO.0b013e328355660b 22766648

[38] Memoli MJ Athota R Reed S Czajkowski L Bristol T Proudfoot K. The natural history of influenza infection in the severely immunocompromised vs nonimmunocompromised hosts. Clin Infect Dis. (2014) 58:214–24. 10.1093/cid/cit725 24186906 PMC3871797

[39] Forns X Navasa M. Liver transplant immunosuppression during the covid-19 pandemic. Gastroenterol Hepatol. (2020) 43:457–63. 10.1016/j.gastre.2020.10.001 32646657 PMC7290227

[40] Rodriguez-Peralvarez M Salcedo M Colmenero J Pons JA. Modulating immunosuppression in liver transplant patients with COVID-19. Gut. (2020). 10.1136/gutjnl-2020-322620. [Epub ahead of print]. 32816964

[41] Fix OK Hameed B Fontana RJ Kwok RM McGuire BM Mulligan DC. Clinical best practice advice for hepatology and liver transplant providers during the COVID-19 pandemic: AASLD expert panel consensus statement. Hepatology. (2020) 72:287–304. 10.1002/hep.31281 32298473 PMC7262242

[42] Bhimraj A Morgan RL Shumaker AH Lavergne V Baden L Cheng VC. Infectious diseases Society of America guidelines on the treatment and management of patients with COVID-19. Clin Infect Dis. (2020). 10.1093/cid/ciaa478. [Epub ahead of print]. 32338708 PMC7197612

[43] Chiotos K Hayes M Kimberlin DW Jones SB James SH Pinninti SG. Multicenter interim guidance on use of antivirals for children with COVID-19/SARS-CoV-2. J Pediatric Infect Dis Soc. (2020). 10.1093/jpids/piaa115. [Epub ahead of print]. 32918548 PMC7543452

[44] National Institutes of Health COVID-19 Treatment Guidelines. (2020). Available online at: https://www.covid19treatmentguidelines.nih.gov/special-populations/children/ (accessed September 29, 2020).

[45] Infectious Disease Society of America Guidelines on the Treatment and Management of Patients wtih COVID-19. (2020). Available online at: https://www.idsociety.org/practice-guideline/covid-19-guideline-treatment-and-management/#toc-7 (accessed September 29, 2020).

[46] Kumar D Manuel O Natori Y Egawa H Grossi P Han SH. COVID-19: a global transplant perspective on successfully navigating a pandemic. Am J Transplant. (2020) 20:1773–9. 10.1111/ajt.15876 32202064 PMC7228301

[47] Boyarsky BJ Po-Yu Chiang T Werbel WA Durand CM Avery RK Getsin SN. Early impact of COVID-19 on transplant center practices and policies in the United States. Am J Transplant. (2020) 20:1809–18. 10.1111/ajt.15915 32282982 PMC7262146

[48] Lembach H Hann A McKay SC Hartog H Vasanth S El-Dalil P. Resuming liver transplantation amid the COVID-19 pandemic. Lancet Gastroenterol Hepatol. (2020) 5:725–6. 10.1016/S2468-1253(20)30187-4 32534603 PMC7289560

[49] Strauss AT Boyarsky BJ Garonzik-Wang JM Werbel W Durand CM Avery RK Liver transplantation in the United States during the COVID-19 pandemic: National and center-level responses. Am J Transplant. (2020). 10.1111/ajt.16373. [Epub ahead of print]. PMC9800484 33107180

[50] UNOS 700 North 4th Street, Richmond, VA 23219. Available online at: www.unos.org (accessed September 29, 2020).

[51] Charnaya O Chiang TP Wang R Motter JD Boyarsky BJ King EA Effects of COVID-19 pandemic on pediatric kidney transplant in the United States. Pediatr Nephrol. (2020):1–9. 10.1007/s00467-020-04764-4. [Epub ahead of print]. PMC7519856 32980942

[52] Federal Communications Commission FCC Approves First Set Of Covid-19 Telehealth Program Applications. Available online at: https://www.fcc.gov/document/fcc-approves-first-set-covid-19-telehealth-program-applications (accessed September 12, 2020).

[53] Sherman CB Said A Kriss M Potluri V Levitsky J Reese PP. In-person outreach and telemedicine in liver and intestinal transplant: a survey of national practices, impact of COVID-19 and areas of opportunity. Liver Transpl. (2020) 26:1354–8. 10.1002/lt.25868 32772459 PMC7436220

[54] Downes KJ Danziger-Isakov LA Cousino MK Green M Michaels MG Muller WJ. Return to school for pediatric solid organ transplant recipients in the United States during the COVID-19 pandemic: expert opinion on key considerations and best practices. J Pediatric Infect Dis Soc. (2020) 9:551–63. 10.1093/jpids/piaa095 32750142 PMC7454776

[55] Feldman AG Beaty BL Curtis D Juarez-Colunga E Kempe A. Incidence of hospitalization for vaccine-preventable infections in children following solid organ transplant and associated morbidity, mortality, and costs. JAMA Pediatr. (2019) 173:260–8. 10.1001/jamapediatrics.2018.4954 30640369 PMC6439884

[56] Russell MR Halnon NJ Alejos JC Salem MM Reardon LC. COVID-19 in a pediatric heart transplant recipient: emergence of donor-specific antibodies. J Heart Lung Transplant. (2020) 39:732–3. 10.1016/j.healun.2020.04.021 32430156 PMC7189188

[57] Heinz N Griesemer A Kinney J Vittorio J Lagana SM Goldner D. A case of an infant with SARS-CoV-2 hepatitis early after liver transplantation. Pediatr Transplant. (2020) 24:e13778. 10.1111/petr.13778 32559354 PMC7323125

[58] Morand A Roquelaure B Colson P Amrane S Bosdure E Raoult D. Child with liver transplant recovers from COVID-19 infection. A case report. Arch Pediatr. (2020) 27:275–6. 10.1016/j.arcped.2020.05.004 32402433 PMC7200359

[59] Bush R Johns F Acharya R Upadhyay K. Mild COVID-19 in a pediatric renal transplant recipient. Am J Transplant. (2020) 20:2942–45. 10.1111/ajt.16003 32406181 PMC7272978

[60] Ravanan R Callaghan CJ Mumford L Ushiro-Lumb I Thorburn D Casey J. SARS-CoV-2 infection and early mortality of wait-listed and solid organ transplant recipients in England: a national cohort study. Am J Transplant. (2020) 20:3008–18. 10.1111/ajt.16247 32780493 PMC7436919

[61] Hendrix MJ Walde C Findley K Trotman R. Absence of apparent transmission of SARS-CoV-2 from two stylists after exposure at a hair salon with a universal face covering policy - Springfield, Missouri, May (2020). MMWR Morb Mortal Wkly Rep. (2020) 69:930–2. 10.15585/mmwr.mm6928e2 32673300

[62] Centers For Disease Control and Prevention Operating Schools during COVID-19: CDC's Considerations. (2020). Available online at: https://www.cdc.gov/coronavirus/2019-ncov/community/schools-childcare/schools.html (accessed September 29, 2020).

[63] American Academy of Pediatrics COVID-19 Planning Considerations: Guidance for School Re-entry (2020). Available online at: https://services.aap.org/en/pages/2019-novel-coronavirus-covid-19-infections/clinical-guidance/covid-19-planning-considerations-return-to-in-person-education-in-schools/ (accessed September 12, 2020).

